# Efficacy of Intravenous Lidocaine for Pain Relief in the Emergency Department: A Systematic Review and Meta-Analysis

**DOI:** 10.3389/fmed.2021.706844

**Published:** 2022-01-17

**Authors:** Junfeng Zhong, Junfeng Hu, Linling Mao, Gang Ye, Kai Qiu, Yuhong Zhao, Shuangyan Hu

**Affiliations:** ^1^Department of Pain Medicine, Shaoxing People's Hospital, Shaoxing, China; ^2^Department of Anesthesiology, Shaoxing Peoples's Hospital, Shaoxing, China

**Keywords:** lidocaine, pain, emergency, analgesic, intravenous

## Abstract

**Objective:**

To compare the efficacy of intravenous (IV) lidocaine with standard analgesics (NSAIDS, opioids) for pain control due to any cause in the emergency department.

**Methods:**

The electronic databases of PubMed, Embase, ScienceDirect, CENTRAL, and Google Scholar were explored from 1st January 2000 to 30th March 2021 and randomized controlled trials (RCTs) comparing IV lidocaine with a control group of standard analgesics were included.

**Results:**

Twelve RCTs including 1,351 patients were included. The cause of pain included abdominal pain, renal or biliary colic, traumatic pain, radicular low back pain, critical limb ischemia, migraine, tension-type headache, and pain of unknown origin. On pooled analysis, we found no statistically significant difference in pain scores between IV lidocaine and control group at 15 min (MD: −0.24 95% CI: −1.08, 0.61 *I*^2^ = 81% *p* = 0.59), 30 min (MD: −0.24 95% CI: −1.03, 0.55 *I*^2^ = 86% *p* = 0.55), 45 min (MD: 0.31 95% CI: −0.66, 1.29 *I*^2^ = 66% *p* = 0.53), and 60 min (MD: 0.59 95% CI: −0.26, 1.44 *I*^2^ = 75% *p* = 0.18). There was no statistically significant difference in the need for rescue analgesics between the two groups (OR: 1.45 95% CI: 0.82, 2.56 *I*^2^ = 41% *p* = 0.20), but on subgroup analysis, the need for rescue analgesics was significantly higher with IV lidocaine in studies on abdominal pain but not for musculoskeletal pain. On meta-analysis, there was no statistically significant difference in the incidence of side-effects between the two study groups (OR: 1.09 95% CI: 0.59, 2.02 *I*^2^ = 48% *p* = 0.78).

**Conclusion:**

IV lidocaine can be considered as an alternative analgesic for pain control in the ED. However, its efficacy may not be higher than standard analgesics. Further RCTs with a large sample size are needed to corroborate the current conclusions.

## Introduction

One of the most frequently encountered complaints in the emergency department (ED) is pain (1). Indeed, early and comprehensive treatment of pain in such a setting is integral to obtaining a high level of patient satisfaction and care. Opioids have been the most frequently prescribed drugs in the ED for pain management (2). Notwithstanding, with the rising incidence of opioid abuse, clinicians are now judicious in the prescription of these drugs in the ED and there is an urgent need for an equally efficacious substitute to manage pain in an urgent setting (3). While the short-term prescription of opioids is not expected to cause drug dependence in itself, there have been apprehensions that the use of opioids in the ED would lead to repeated opioid use in the future, thereby acting as a possible trigger for drug abuse (4, 5). Furthermore, the availability of an alternative drug to opioids with proven efficacy and safety profile would be beneficial in a special group of individuals like older adults, drug addicts, patients with prior history of drug abuse or mental health issues, and long-term opioid users (6, 7).

Lidocaine is a widely used amide local anesthetic that acts by blocking Na channels in the central and peripheral neurons of the nociceptive pathway (8). While lidocaine is commonly used for nerve blocks and infiltration anesthesia, the drug also acts an analgesic when administered intravenously (IV) (9). Several RCTs have shown that the use of IV lidocaine significantly reduces postoperative opioid consumption, decreases pain intensity, and shortens hospital stay in surgical patients (10, 11). A meta-analysis of 26 studies by Zhu et al. (12) have demonstrated that IV lidocaine is effective for the management of patients with neuropathic pain. Another review by Lee et al. (13) has shown that IV lidocaine can be used for refractory cancer pain wherein standard analgesic agents are ineffective. While there have been several trials assessing the efficacy of lidocaine for pain relief, its use in an emergency setting is sparsely reported. In a review article published in 2014, Golzari et al. (14) have summarized evidence on the use of lidocaine in an ED setting. The authors noted that lidocaine has been used via several routes including IV, topical, subcutaneous and intra-articular, and for a variety of different indications in the ED but with variable efficacy. Furthermore, there is no clarity on how each route of administration of lidocaine differs from the other.

To the best of our knowledge, to date, only a few systematic reviews have assessed the efficacy of IV lidocaine in the ED. Two studies by Silva et al. (15) and Masic et al. (16) have analyzed the efficacy of IV lidocaine for all pain indications in the ED while another review by Miller et al. (17) assessed the analgesic effect of IV lidocaine for renal colics. However, a major limitation of these reviews is that they could include only a limited number of randomized controlled trials (RCTs) and some even combined evidence with case reports and case series. Furthermore, no meta-analysis could be carried out in any of these reviews. Thus, no level-1 high-quality evidence on the efficacy of lidocaine as an alternative non-opioid drug is available for clinicians managing patients in an emergency setting. In view of such deficiency in literature, the current study attempted to assess the efficacy and safety of IV lidocaine vis-à-vis standard analgesics like opioids and non-steroidal anti-inflammatory drugs (NSAIDs) for pain control in the ED.

## Materials and Methods

The PRISMA statement (Preferred Reporting Items for Systematic Reviews and Meta-analyses) (18) and the Cochrane Handbook for Systematic Reviews of Intervention (19) were followed during the conduct of this review. The research question to be answered was: What is the efficacy of IV lidocaine vs. standard analgesics for pain management in the emergency department?

### Literature Search

A search strategy was designed with the help of the medical librarian wherein the electronic databases of PubMed, Embase, ScienceDirect, CENTRAL, and Google Scholar were explored. The search limits were set from inception to 26th October 2021. We also search clinicaltrials.gov for any ongoing trial. However, excluded databases with articles preprints that have not been peer-reviewed. A mix of MeSH and free keywords used for the literature search included: “lidocaine,” “lignocaine,” “emergency,” “pain,” and “analgesic.” The search strings used were “(((lidocaine) OR (lignocaine)) AND (analgesic)) AND (emergency)” and “(((lidocaine) OR (lignocaine)) AND (pain)) AND (emergency).” Two reviewers carried out the electronic search independent of each other. The primary search results were assessed initially by their titles and abstracts to identify citations requiring full-text analysis. The full texts of the articles were reviewed by the two reviewers independently based on the inclusion and exclusion criteria. Any disagreements were resolved by discussion. Furthermore, we also hand-searched the bibliography of included studies for any missed references.

### Inclusion Criteria

Eligibility criteria for this review were structured using the PICOS (Population, Intervention, Comparison, Outcome, and Study design) framework. Details are as follows:

**Population**: Patients reporting to the ED for any kind of pain

**Intervention**: IV lidocaine in any dose

**Comparison**: NSAIDs or opioids via any route and any dose (Control group)

**Outcomes**: Pain scores in the first 60 min, use of rescue analgesics, and/or side-effects of the drugs.

**Study design**: Randomized controlled trials (RCTs) only

**Exclusion criteria were**: (1) Studies using IV lidocaine as an adjuvant to other analgesics (2) Studies not using a comparative analgesic drug or not using NSAIDS/opioids as a comparative drug (3) Non-RCTs and uncontrolled studies (4) Studies not reporting relevant outcomes (5) Editorials, review articles, and non-English language studies.

### Data Extraction and Risk of Bias Assessment

Two reviewers extracted data independently using a data extraction sheet. Data regarding the first author, publication year, study location, cause of pain, inclusion criteria, sample size, mean age, gender details, the dose of IV lidocaine, type and dose of the comparative drug, and study outcomes were extracted. Primarily, we aimed to analyze the difference in pain scores in the first 60 min after drug administration. Secondary outcomes of interest were the need for rescue analgesics in the two groups and side-effects associated with the interventions. A descriptive analysis was carried out if sufficient data were not available for a meta-analysis. The corresponding authors were not contacted for any missing data.

We used the recent Cochrane Collaboration's risk of bias assessment tool-2 to assess the quality of included RCTs (19). This was done by two reviewers independently. The following five domains were used for quality assessment: randomization process, deviation from intended intervention, missing outcome data, measurement of outcomes, and selection of reported result. Based on the risk of bias in individual domains, the overall bias was marked as “high risk”, “some concerns,” or “low risk.” Any disagreements related to data extraction or quality assessment were resolved by discussion. We also assessed the certainty of the evidence using the Grading of Recommendations Assessment, Development, and Evaluation (GRADE) tool using the GRADEpro GDT software [GRADEpro Guideline Development Tool. McMaster University, 2020 (developed by Evidence Prime, Inc.)].

### Statistical Analysis

“Review Manager” (RevMan, version 5.3; Nordic Cochrane Centre [Cochrane Collaboration], Copenhagen, Denmark; 2014) was used for the meta-analysis. Pain scores at different time intervals were summarized using mean Difference (MD) with 95% confidence intervals (CI). Need for rescue analgesics and side-effects were summarized using odds ratios (OR) with 95% CI. For studies reporting data only in graphical format, Engauge Digitizer Version 12.1 was used to extract data. Median, range and interquartile range data was converted into mean and standard deviation (SD) when required using the method of Wan et al. (20). Heterogeneity was assessed using the *I*^2^ statistic. *I*^2^ values of 25–50% represented low, values of 50–75% medium, and more than 75% represented substantial heterogeneity. However, irrespective of the heterogeneity we preferred to use a random-effects model for our meta-analysis as the included studies were conducted on different populations with significant methodological heterogeneity. Due to a limited number of studies in the meta-analysis (<10), funnel plots were not used to assess publication bias. Sub-group analyses based on the type of pain and type of comparative drug (opioid/NSAID) were also performed if there were at least 2 studies in each subgroup. We grouped studies reporting pain of abdominal origin (including biliary colic, renal colic) and musculoskeletal pain separately to separately assess the efficacy of IV lidocaine for each pain type.

## Results

### Details of Included Studies

**Figure 1:**
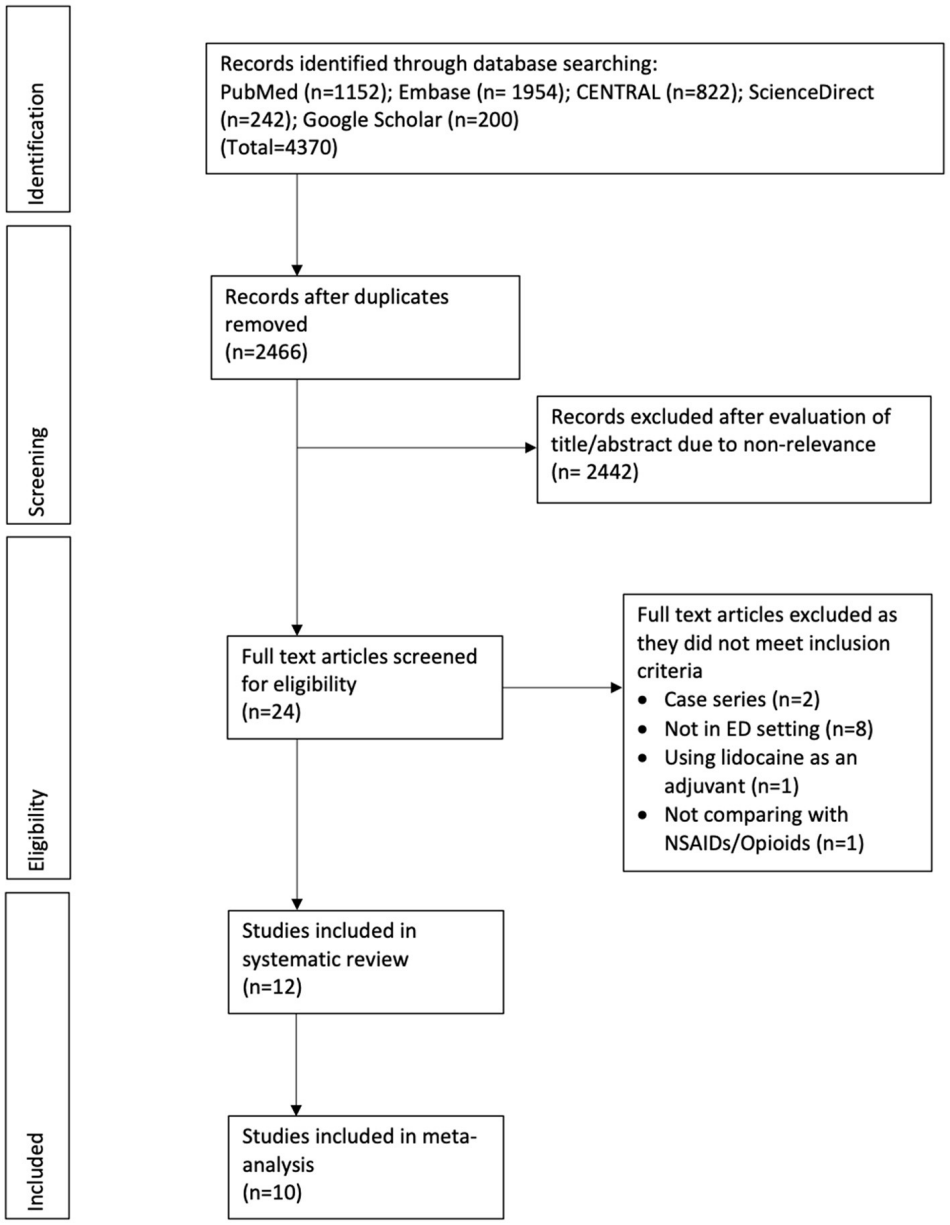
Study flow chart.

The number of search results at each stage is summarized in Figure 1. We reviewed a total of 2,466 unique records. Of these 2,442 were excluded based on title and abstract screening. Twenty-four articles were analyzed by their full-text and 12 were excluded with reasons. A total of 12 RCTs fulfilled the inclusion criteria and were analyzed in this review (21–32). The agreement between the two reviewers on inclusion of the studies was high (kappa 0.9). Baseline details extracted from the studies are presented in Table 1. A total of 1,351 patients were included in these 12 RCTs. All included trials were conducted either in Iran, Turkey or the USA. The cause of pain varied across studies and included abdominal pain, renal or biliary colic, traumatic pain, radicular low back pain, critical limb ischemia, migraine, tension-type headache, and pain of unknown origin. In studies using a fixed dose of lidocaine the dosage ranged from 100 to 150 mg while in studies using weight-based dosage, it ranged from 1.5 to 2 mg/kg. IV morphine was the most common comparative drug while hydromorphone, fentanyl, and ketorolac (all administered IV) were used in the control group in one trial each. Two recent studies used IV dexketoprofen in the control group. Two trials of Motamed et al. (29) and Forouzan et al. (31) did not report pain scores as mean and SD but reported the number of patients with mild, moderate, and severe pain at different time intervals. The secondary outcome in both trials was treatment failure defined as lack of 3-point reduction of pain scores on Visual Analog Scale (VAS). Analyzing their results descriptively, both studies did not find any difference in the number of patients with different pain intensities at 15 and 30 min. While Motamed et al. (29) noted higher failure rates with IV lidocaine, Forouzan et al. (31) found higher failure rates in the control group. Another study of Akbas et al. (21) reported only change in pain scores at different time intervals. The authors noted significantly greater reduction of pain scores with IV lidocaine at 20, 30, 60, 90, and 120 min as compared to IV dexketoprofen.

**Table 1:** Details of included studies.

**Study**	**Location**	**Cause** **of pain**	**Inclusion** **criteria**	**IV Lidocaine** **dose**	**Control group drug**	**Sample** **size**		**Mean** **age (years)**		**Male** **gender (%)**		**Study conclusions**
						**L**	**C**	**L**	**C**	**L**	**C**	
Gur et al. (22)	Turkey	Migraine	Adult patients attending ED with migraine attacks with at least a 1-year history of migraine	1.5 mg/kg bolus followed by 1 mg/kg/h infusion for 30 min and 0.5 mg/kg/h for next 30 min	Normal saline bolus followed by infusion of IV dexketoprofen 50 mg	50	50	43 [33–55]	37 [33–54]	38	46	IV lidocaine can be an alternative modality to manage migraine headaches
Akbas et al. (21)	Turkey	Tension-type headache	Adult patients attending ED with episodic tension-type headache	1.5 mg/kg	IV dexketoprofen 50 mg	60	60	43 [29–50.3]	43 [30–54]	66.7	58.3	IV lidocaine can be useful to manage tension-type headaches
Akhgar et al. (26)	Iran	Suspected biliary colic	Adult patients attending ED with right upper quadrant pain with severity >5 on NRS	100 mg	IV morphine 5 mg	51	53	44.13 ± 14.98	44.3 ± 12.78	49	45	No significant difference between lidocaine and morphine for pain relief
Motov et al. (25)	USA	Renal colic	Adult patients attending ED with acute flank pain, abdominal pain, or back pain suspected to be due to renal colic	1.5 mg/kg	IV ketorolac 30 mg	50	50	39.34 ± 10.95	42.34 ± 10.47	54	56	IV ketorolac was superior to IV lidocaine for pain relief
Chinn et al. (27)	USA	Acute abdominal	Adult patients with acute (<7 days) and severe (requiring IV opioids) abdominal pain	120 mg	IV hydromorphone 1 mg	77	77	42 ± 12	40 ± 13	30	43	IV hydromorphone was superior to IV lidocaine for pain control
Clattenburg et al. (28)	USA	Unknown cause	Adult patients with pain of severity ≥7 on NRS	1.5 mg/kg bolus and 1.5 mg/kg infusion over 50 min	IV morphine based on physicians discretion	16	16	50 [36.5–59.5]	45.5 [34–59.5]	50	44	No significant difference between lidocaine and morphine for pain relief
Farahmand et al. (30)	Iran	Traumatic	Adult patients with acute extremity injury and pain score of >4 on NRS	150 mg	IV morphine 10 mg	25	25	31.4 ± 8.73	31.16 ± 8.7	76	80	IV lidocaine is not superior to IV morphine for pain control
Motamed and Verki (29)	Iran	Renal colic	Adult patients with acute colicky flank pain	1.5 mg/kg	IV fentanyl 1.5 μg/kg	45	45	39.08 ± 6.64	34.08 ± 9.49	86.7	93.3	No difference in pain scores between the two drugs but higher treatment failure with IV lidocaine
Forouzan et al. (31)	Iran	Traumatic	Patients aged 15–65 with fracture of long bones in moderate to severe pain	1.5 mg/kg	IV morphine 0.1 mg/kg	140	140	31.47 ± 12.31	33.53 ± 13.16	50.2	49.8	IV lidocaine can be a reasonable alternative to morphine for pain management
Vahidi et al. (23)	Iran	Critical limb ischemia	Patients aged >15 years diagnosed with critical limb ischemia based on their clinical findings	2 mg/kg	IV morphine 0.1 mg/kg	20	20	63.95 ± 11.6	63.8 ± 12.2	45.8	54.2	IV lidocaine was found to be better as compared to morphine for pain relief
Tanen et al. (32)	USA	Radicular low back pain	Patients aged 15–55 years with radicular lower back pain	100 mg	IV ketorolac 30 mg	21	20	36 ± 10	39 ± 12	52.4	70	IV lidocaine failed to clinically alleviate pain as compared to ketorolac
Soleimanpour et al. (24)	Iran	Renal colic	Adult patients with renal colicky pain	1.5 mg/kg	IV morphine 0.1 mg/kg	120	120	35.23 ± 12.37	37.71 ± 11.08	71.7	75	IV lidocaine causes significant reduction of renal colic pain

*IV, intravenous; L, lidocaine group; C, control group; ED, emergency department*.

### Meta-Analysis

**Figure 2:**
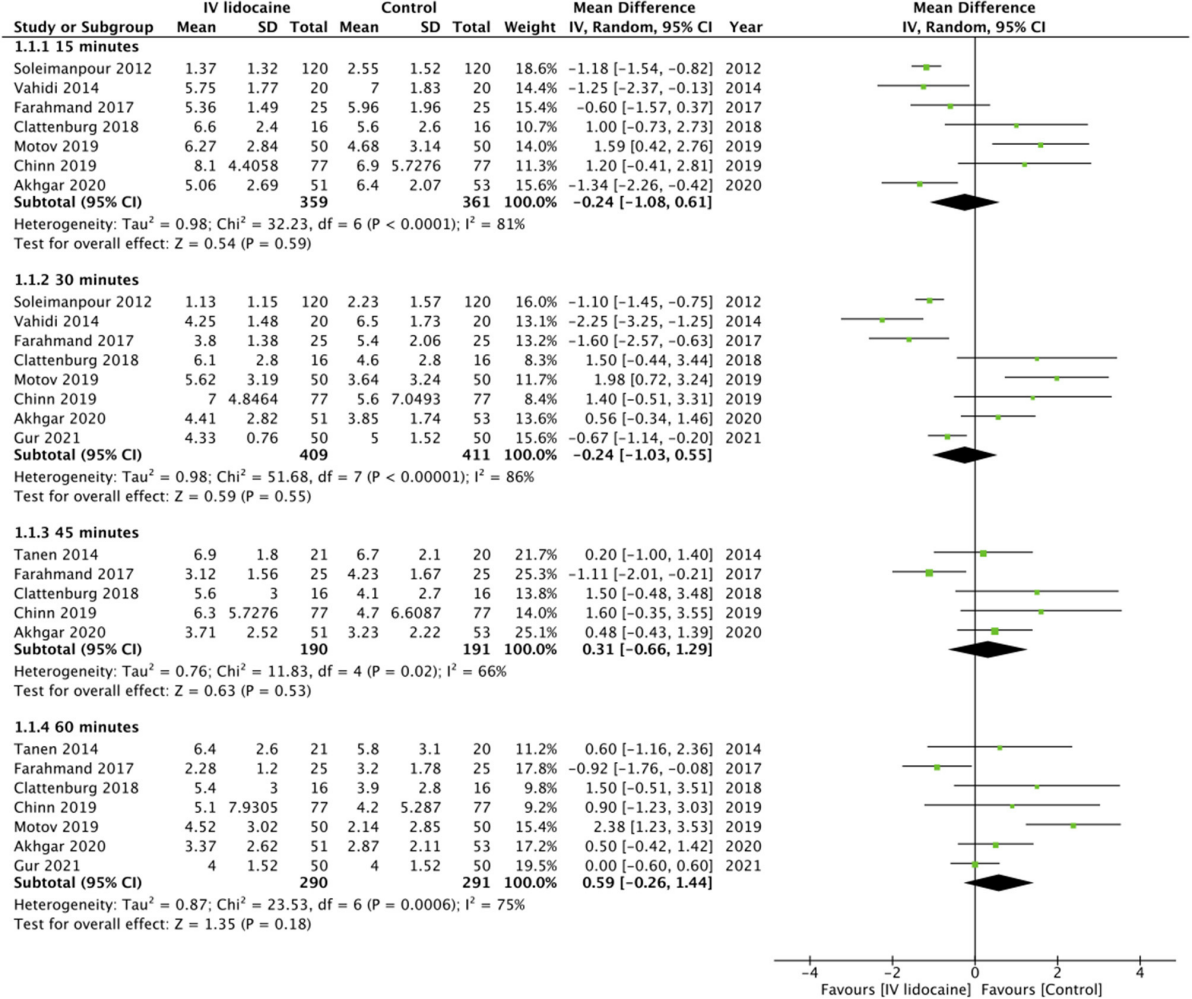
Meta-analysis of pain scores at different time points between IV lidocaine and control groups.

On pooled analysis, we found no statistically significant difference in pain scores between IV lidocaine and control group at 15 min (MD: −0.24 95% CI: −1.08, 0.61 *I*^2^ = 81% *p* = 0.59), 30 min (MD: −0.24 95% CI: −1.03, 0.55 *I*^2^ = 86% *p* = 0.55), 45 min (MD: 0.31 95% CI: −0.66, 1.29 *I*^2^ = 66% *p* = 0.53), and 60 min (MD: 0.59 95% CI: −0.26, 1.44 *I*^2^ = 75% *p* = 0.18) (Figure 2). GRADE assessment of the certainty of the evidence was “moderate” (Supplementary Table 1). On subgroup analysis based on the origin of pain, there was no statistically significant difference in pain scores between the two groups for studies on abdominal pain at any time point (Table 2). However, on analysis of just two studies on musculoskeletal pain, we noted significantly lower pain scores with IV lidocaine as compared to the control group. Similarly, subgroup analysis based on the type of control drug, we noted no difference in pain scores at 30 and 60 min between IV lidocaine and opioids or NSAIDs (Table 2).

**Table 2:** Results of sub-group analysis.

**Outcome**	**Sub-group**	**Number of studies**	**Effect size**
Pain score 15 min	Abdominal Musculoskeletal	4 2	MD: −0.05 95% CI: −1.41, 1.31 *I*^2^ = 89% *p* = 0.94 MD: −0.88 95% CI: −1.61, −0.15 *I*^2^ = 0% *p* = 0.02
Pain score 30 min	Abdominal Musculoskeletal	4 2	MD: −0.60 95% CI: −0.98, 2.19 *I*^2^ = 91% *p* = 0.45 MD: −1.92 95% CI: −2.61, −1.22 *I*^2^ = 0% *p* < 0.00001
	Opioids NSAIDs	6 2	MD: −0.48 95% CI: −1.46, 0.50 *I*^2^ = 84% *p* = 0.34 MD: 0.59 95% CI: −2.01, 3.18 *I*^2^ = 93% *p* = 0.66
Pain score 45 min	Abdominal Musculoskeletal	2 2	MD: 0.69 95% CI: −0.17, 1.56 *I*^2^ = 3% *p* = 0.11 MD: −0.52 95% CI: −1.80, 0.76 *I*^2^ = 66% *p* = 0.43
Pain score 60 min	Abdominal Musculoskeletal	3 2	MD: 1.28 95% CI: −0.06, 2.62 *I*^2^ = 69% *p* = 0.06 MD: −0.36 95% CI: −1.80, 1.07 *I*^2^ = 57% *p* = 0.62
	Opioids NSAIDs	5 2	MD: 0.80 95% CI: −0.51, 2.11 *I*^2^ = 82% *p* = 0.23 MD: 0.06 95% CI: −0.50, 0.63 *I*^2^ = 0% *p* = 0.83
Rescue analgesics	Abdominal Musculoskeletal	3 2	OR: 2.44 95% CI: 1.44, 4.15 *I*^2^ = 0% *p* = 0.0009 OR: 0.45 95% CI: 0.05, 4.18 *I*^2^ = 54% *p* = 0.48
	Opioids NSAIDs	5 2	OR: 1.68 95% CI: 0.77, 3.63 *I*^2^ = 46% *p* = 0.19 OR: 1.00 95% CI: 0.50, 2.00 *I*^2^ = 0% *p* = 0.01

*MD, mean difference; OR, odds ratio; CI, confidence intervals; NSAIDs, Non-steroidal anti-inflammatory drugs*.

**Figure 3:**
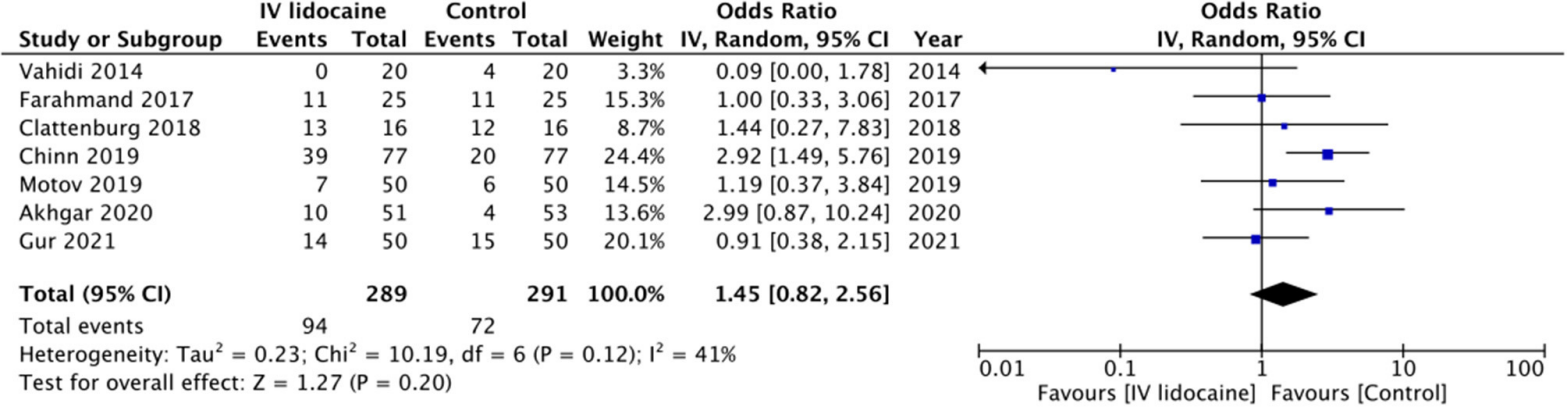
Meta-analysis of need for rescue analgesics between IV lidocaine and control groups.

There was no statistically significant difference in the need for rescue analgesics between the two groups (OR: 1.45 95% CI: 0.82, 2.56 *I*^2^ = 41% *p* = 0.20) (Figure 3). The certainty of evidence based on GRADE was “low” (Supplementary Table 1). But on subgroup analysis, the need for rescue analgesics was significantly higher with IV lidocaine in studies on abdominal pain but not for musculoskeletal pain (Table 2). Subgroup analysis based in the type of control group drug revealed no difference between the two groups.

**Figure 4:**
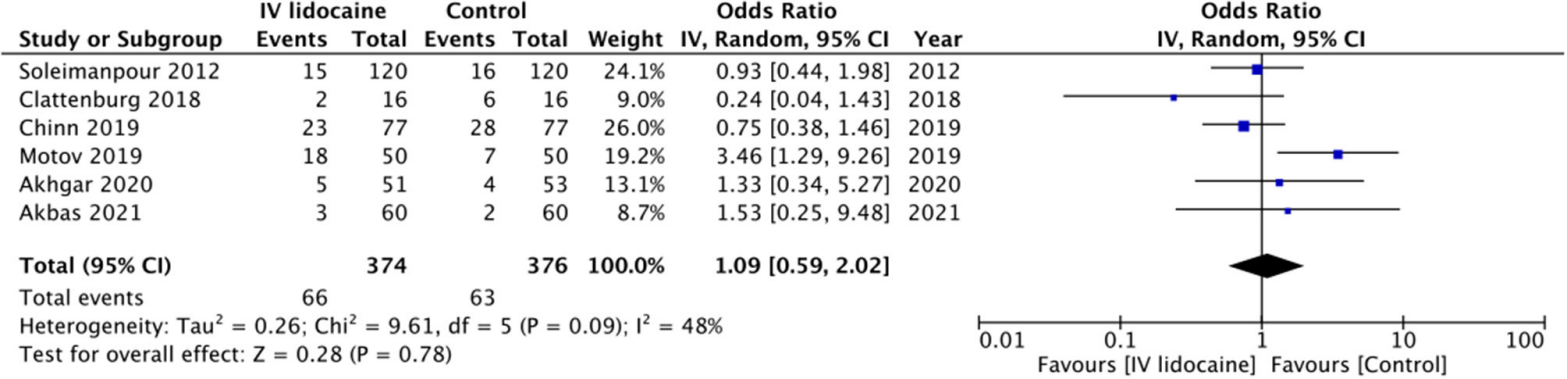
Meta-analysis of incidence of side-effects between IV lidocaine and control groups.

Details of side-effects reported by included studies are presented in Table 3. On meta-analysis, there was no statistically significant difference in the incidence of side-effects between the two study groups (OR: 1.09 95% CI: 0.59, 2.02 *I*^2^ = 48% *p* = 0.78) (Figure 4). The certainty of evidence based on GRADE was “moderate” (Supplementary Table 1).

**Table 3:** Details of adverse events reported by included studies.

**Study**	**Lidocaine**	**Control**
Akhgar et al. (26)	Dizziness (9.8%)	Vomiting (7.5%)
Motov et al. (25)	Dizziness Nausea/Vomiting Perioral numbness Tinnitus Headache Epigastric pain Drowsiness	Dizziness Nausea/Vomiting Headache Epigastric pain
Chinn et al. (27)	Dizziness (5%) Drowsiness (8%) Headache (8%) Nausea (12%) Pruritis (1%)	Dizziness (14%) Drowsiness (4%) Headache (3%) Nausea (13%) Pruritis (2%)
Clattenburg et al. (28)	Perioral numbness (6.3%) Nausea (6.3%)	Nausea (25%) Pruritis (6.3%) Bradycardia (6.3%)
Farahmand et al. (30)	Vomiting (4%)	Vomiting (4%)
Soleimanpour et al. (24)	Perioral numbness (2.5%) Transient dizziness (8.3%) Dysarthria (1.7%)	Hypotension (2.5%) Vertigo (1.7%) Nausea (7.5%) Vomiting (1.6%)

*Figures in parenthesis indicate percentage of patients experiencing the adverse event (where data was available)*.

### Risk of Bias

The risk of bias summary of the included studies is presented in Table 4. Nine studies were considered to have a “low” overall risk of bias. The study of Chinn et al. (27) was unblinded and therefore considered to have a “high” risk of bias for deviation from intended intervention and measurement of outcomes. The two trials (29, 31) not included in the meta-analysis were considered to have a “high” overall risk of bias due to the selection of reported results. These trials did not use standard methods of reporting pain outcomes and did not present complete outcome data.

**Table 4:** Risk of bias in included studies.

**Study**	**Randomization process**	**Deviation from intended intervention**	**Missing outcome data**	**Measurement of outcomes**	**Selection of reported result**	**Overall risk of bias**
Gur et al. (22)	Low risk	Low risk	Low risk	Low risk	Low risk	Low risk
Akbas et al. (21)	Low risk	Low risk	Low risk	Low risk	Low risk	Low risk
Akhgar et al. (26)	Low risk	Low risk	Low risk	Low risk	Low risk	Low risk
Motov et al. (25)	Low risk	Low risk	Low risk	Low risk	Low risk	Low risk
Chinn et al. (27)	Some concerns	High risk	Low risk	High risk	Low risk	High risk
Clattenburg et al. (28)	Low risk	Low risk	Low risk	Low risk	Low risk	Low risk
Farahmand et al. (30)	Low risk	Low risk	Low risk	Low risk	Low risk	Low risk
Motamed and Verki (29)	Low risk	Low risk	Low risk	Low risk	High risk	High risk
Forouzan et al. (31)	Low risk	Low risk	Low risk	Low risk	High risk	High risk
Vahidi et al. (23)	Low risk	Low risk	Low risk	Low risk	Low risk	Low risk
Tanen et al. (32)	Low risk	Low risk	Low risk	Low risk	Low risk	Low risk
Soleimanpour et al. (24)	Low risk	Low risk	Low risk	Low risk	Low risk	Low risk

## Discussion

Treatment of pain is an important yet complex problem in an ED. While managing this symptom, the clinician has to not only make a quick diagnosis of the origin of pain but also take into account the past medical history, drug history, and severity of the problem to prescribe an optimal analgesic. On account of several apprehensions amongst healthcare professionals regarding the opioid epidemic (33), research in the past decade has focussed on providing an optimal and safe non-opioid analgesic for routine use in the ED (34). The 2017 policy statement by the American College of Emergency Physicians also recommends that the first intervention for acute pain in the ED should be a non-opioid drug (35). Indeed, the second most commonly prescribed analgesics are NSAIDs and their use is gradually increasing for the management of pain in an emergency setting (36). According to a systematic review and meta-analysis by Pathan et al. (37), the efficacy of NSAIDs is equivalent to opioids for the management of renal colic and they can be used as a suitable alternative for the management of colicky pain in the ED. Another recent study by Yin et al. (38) has shown that NSAIDs are most suitable for the management of musculoskeletal pain. Despite their efficacy, there have been concerns regarding the use of NSAIDS for severe pain and the associated side effects of these drugs. Gastrointestinal bleeding/ulceration, renal injury, and platelet inhibition are known adverse events linked with NSAIDs (39). In this context, an alternative drug like IV lidocaine can expand the spectrum of medications available to an emergency physician for the management of pain.

As seen in our systematic review, with the availability of only 12 trials, IV lidocaine has not been widely researched for pain management in the ED. However, the drug has been used on a wide range of patients including both musculoskeletal and abdominal pain. On pooled analysis of the trials, we found no difference in pain scores between patients receiving IV lidocaine or standard analgesics at different time points in the first hour of drug administration. Interesting to note was that in the first 30 min, there was a significant but small reduction of pain scores with IV lidocaine in studies on musculoskeletal pain but not for abdominal pain. Nevertheless, this should be interpreted with the concept of minimum clinically important difference (MCID). In 1989, Jaeschke et al. (40) bought forward the principle of MCID, which was defined as “the smallest difference in score in the domain of interest which participants perceive as beneficial and which would mandate, in the absence of troublesome side effects and costs, a change in the patient's management.” This concept stresses the fact that the difference in pain scores achieved by the drug should be clinically relevant to the patient even if the MD is statistically significant (41). Considering the MD in our meta-analysis was very small, it may not be clinically relevant. Furthermore, we also noted that there was no difference in pain outcomes between IV lidocaine vs. opioid as well as IV lidocaine vs. NSAIDs. However, there were only limited number of studies in our subgroup analyses and the results should be interpreted cautiously. Another important parameter of assessing pain in any trial is the need for rescue analgesics which is a surrogate marker for the amount of pain experienced by the patient. We found that the need for rescue analgesics was higher at 33.4% in the IV lidocaine group as compared to 23.6% in the control group but with a non-significant difference. However, one has to note that the 95% CI of the meta-analysis on the need for rescue analgesics was wide (0.86–3.08) with the lower limit close to 1 and upper limit indicating a 3-fold increased use of rescue analgesics with IV lidocaine. Thus, while pain scores may not differ between the two groups, there was a tendency of increased use of rescue analgesics in the IV lidocaine group which affected the pain scores. Owing to the limited number of studies reporting data on the need for rescue analgesics and the wide 95% CI the overall certainty of the evidence was downgraded and was deemed to be “low.” On subgroup analysis, it was obvious that the need for rescue analgesics was significantly high in studies on abdominal pain with a similar albeit non-significant tendency in studies on musculoskeletal pain as well. However, we noted no difference in the need for rescue analgesics based on the type of control drug indicating similar efficacy of lidocaine as compared to both groups of analgesic drugs. However, this must be interpreted with caution due to limited number of studies in the subgroup analysis and further trials are needed to strengthen this comparison.

For comparing our results with prior literature, it can be noted that the use of IV lidocaine has been most commonly reported in a surgical setting (42). While the majority of studies on surgical patients indicate that lidocaine is an effective analgesic for postoperative pain control (10, 11), few trials have indicated that systemic lidocaine may offer no beneficial effect in the postoperative period (43, 44). However, it is important to note that majority of the studies assessing the efficacy of IV lidocaine in a surgical setting have compared the drug with placebo or used IV lidocaine as an adjunct to a baseline analgesic. Therefore, in the absence of a comparator drug, the analgesic property of IV lidocaine would be more evident. The results of the only trial assessing the efficacy of lidocaine as an adjunct in an emergency setting have shown that IV lidocaine is an effective adjuvant to morphine for managing renal colic pain in the ED (45). However, literature is scarce for direct comparison between IV lidocaine and other analgesics. In a small trial, Wu et al. (46) have shown that morphine is superior to IV lidocaine for managing post-amputation pain.

The use of IV lidocaine as a potential analgesic in the ED is accompanied by its inherent complications. Due to the associated cardiotoxicity and neurotoxicity, IV lidocaine should be cautiously used in patients with comorbidities like heart block, cardiac failure, or epilepsy (47). Furthermore, owing to its narrow therapeutic index constant ECG and blood pressure monitoring are needed following IV administration of lidocaine. Toxicity with lidocaine usually manifests as numbness of the tongue, metallic taste, drowsiness, and tinnitus. At higher doses, patients may experience visual disturbances, muscle twitching, and seizures (48). These effects are largely based on the dosage of the drug use. While the exact dosage of IV lidocaine for pain control is not clear, usually a weight-based dose of 1.5–2 mg/kg is utilized. A similar dose was used by the majority of studies in our review. Dizziness, perioral numbness, nausea were commonly noted in the lidocaine arm of the trials (49). However, there was no statistically significant difference in the incidence of side-effects between the two study groups and none of the studies reported any serious adverse events with lidocaine like bradycardia, hypotension, or seizures.

Our review has some limitations. Foremost, the number of trials available in the meta-analysis was not high. Two studies had to be excluded from the analysis due to differences in reporting of outcomes. Secondly, there was significant heterogeneity in the included studies concerning the diagnosis, dosage of lidocaine, and comparative drug used. An attempt was made to explore this heterogeneity using a subgroup analysis for the type of pain and type of control group analgesic. However, this reduced the power of the analysis. Thirdly, not all studies were high-quality and free of bias. The certainty of evidence provided by the review was not high and ranged from moderate-low. Lastly, all the included trials were conducted in just two countries. This significantly limits the generalizability of the results of our review.

Nevertheless, our study is the first meta-analysis to assess the efficacy and safety of IV lidocaine for pain control in an emergency setting. Unlike prior reviews (15, 16), we included only RCTs to present the best possible evidence to the readers. Subgroup analysis and GRADE assessment was done to provide clarity on the results.

To conclude, our results indicate that IV lidocaine can be considered as an alternative analgesic for pain control in the ED. However, its efficacy may not be higher than standard analgesics. Further RCTs with a large sample size are needed to corroborate the current conclusions.

## Data Availability Statement

The raw data supporting the conclusions of this article will be made available by the authors, without undue reservation.

## Author Contributions

JZ, JH, and LM: conceptualized and designed the study and analyzed the data. GY, KQ, and YZ: literature search. GY, KQ, and SH: writing—original draft preparation. YZ and SH: writing—reviewing and editing. All authors read and approved the final manuscript.

## Funding

This study was funded by Project of Shaoxing Municipal Science and Technology (2018C30078).

## Conflict of Interest

The authors declare that the research was conducted in the absence of any commercial or financial relationships that could be construed as a potential conflict of interest.

## Publisher's Note

All claims expressed in this article are solely those of the authors and do not necessarily represent those of their affiliated organizations, or those of the publisher, the editors and the reviewers. Any product that may be evaluated in this article, or claim that may be made by its manufacturer, is not guaranteed or endorsed by the publisher.
