# Supplementary material for: Efficacy of Intravenous Lidocaine for Pain Relief in the Emergency Department: A Systematic Review and Meta-Analysis
Source: Front Med (Lausanne). 2022 Jan 17;8:706844. doi: 10.3389/fmed.2021.706844 (PMC8801430; doi:10.3389/fmed.2021.706844)
Supplement: Supplementary file 1 [file Table_1.DOCX]

| **Supplementary Table 1: GRADE assessment of evidence** | | | | | | | | | | | |
| --- | --- | --- | --- | --- | --- | --- | --- | --- | --- | --- | --- |
| **Certainty assessment** | | | | | | | **Summary of findings** | | | | |
| **Participants  (studies) Follow up** | **Risk of bias** | **Inconsistency** | **Indirectness** | **Imprecision** | **Publication bias** | **Overall certainty of evidence** | **Study event rates (%)** | | **Relative effect (95% CI)** | **Anticipated absolute effects** | |
|  |  |  |  |  |  |  | **With Control** | **With IV lidocaine** |  | **Risk with Control** | **Risk difference with IV lidocaine** |
| **Pain - 15 minutes** | | | | | | | | | | | |
| 720 (7 RCTs) | serious ^a^ | not serious | not serious | not serious | none | ⨁⨁⨁◯ MODERATE | 361 | 359 | - | The mean pain - 15 minutes was **0** | MD **0.24 lower** (1.08 lower to 0.61 higher) |
| **Pain - 30 minutes** | | | | | | | | | | | |
| 820 (8 RCTs) | serious ^a^ | not serious | not serious | not serious | none | ⨁⨁⨁◯ MODERATE | 411 | 409 | - | The mean pain - 30 minutes was **0** | MD **0.24 lower** (1.03 lower to 0.55 higher) |
| **Pain - 45 minutes** | | | | | | | | | | | |
| 381 (5 RCTs) | serious ^a^ | not serious | not serious | not serious | none | ⨁⨁⨁◯ MODERATE | 191 | 190 | - | The mean pain - 45 minutes was **0** | MD **0.31 higher** (0.66 lower to 1.29 higher) |
| **Pain - 60 minutes** | | | | | | | | | | | |
| 581 (7 RCTs) | serious ^a^ | not serious | not serious | not serious | none | ⨁⨁⨁◯ MODERATE | 291 | 290 | - | The mean pain - 60 minutes was **0** | MD **0.59 higher** (0.26 lower to 1.44 higher) |
| **Rescue analgesics** | | | | | | | | | | | |
| 580 (7 RCTs) | serious ^a^ | not serious | not serious | serious ^b^ | none | ⨁⨁◯◯ LOW | 72/291 (24.7%) | 94/289 (32.5%) | **OR 1.45** (0.82 to 2.56) | 237 per 1,000 | **75 more per 1,000** (from 35 fewer to 210 more) |
| **Side-effects** | | | | | | | | | | | |
| 750 (6 RCTs) | serious ^a^ | not serious | not serious | not serious | none | ⨁⨁⨁◯ MODERATE | 63/376 (16.8%) | 66/374 (17.6%) | **OR 1.09** (0.59 to 2.02) | 193 per 1,000 | **12 more per 1,000** (from 61 fewer to 122 more) |

**CI:** Confidence interval; **MD:** Mean difference; **OR:** Odds ratio

#### Explanations

a. Due to high risk of bias in the study of Chinn et al

b. wide 95% CI with results unstable on subgroup analysis
